# A case of endocervical adenocarcinoma of the gastric type that was repeatedly misdiagnosed: A case report and literature review

**DOI:** 10.1097/MD.0000000000033682

**Published:** 2023-05-26

**Authors:** Shurong Zhang, Guoyu Jin, Feier Ding, Junlei Zhang, Qiuju Li, Guoyun Wang, Changzhong Li, Chunrun Yang

**Affiliations:** a Department of Gynecological Oncology, Shandong Provincial Hospital, Shandong University, Jinan, China; b Department of Gynecological Oncology, Shandong University of Traditional Chinese Medicine, Jinan, China; c Department of Gynecological Oncology, Shandong Provincial Hospital, Jinan, China.

**Keywords:** adenocarcinoma, case report, endocervical, gastric-type, human papillomavirus

## Abstract

Gastric-type endocervical adenocarcinoma (GAS) is non-human papillomavirus-associated cervical cancer and the location of the lesions is in the cervical canal mostly.

**Patient concerns:**

Vaginal discharge is mistakenly thought to be caused by uterine fibroids. Misdiagnosis leads to disease progression.

**Diagnoses:**

Magnetic resonance imaging is an auxiliary tool and pathology is the gold standard for the diagnosis.

**Interventions:**

Surgery and supplementary radiotherapy and chemotherapy ± targeted therapy are the main treatment methods.

**Outcomes:**

GAS with high malignant degree poor prognosis and insidious development, tends to develop toward the cervical canal and is lack of specific tumor markers, so it is easy to misdiagnosis and missed diagnosis.

**Lessons:**

This case highlights the importance of improving the understanding of GAS. And when patients perform vaginal discharge, cervical canal hypertrophy, and cervical cancer screening negative, clinicians ought to be highly alert to GAS.

## 1. Introduction

Cervical cancer is the fourth most frequent malignant tumor among women internationally, with an estimated 604,000 new cases and 342,000 fatal cases in 2020.^[1]^ Among cervical cancer, more are associated with human papillomavirus (HPV) infection, while a few are non-HPV-related. Due to the highly effective tertiary prevention measures, the incidence of HPV-related cervical cancer has been decreasing year by year. However, some cervical cancer patients with HPV negative are easily forgotten and missed, which is terrible. Therefore, it is a new challenge to identify and diagnose non-HPV-associated cervical cancer in the clinic. Endocervical adenocarcinomas (AC) can be divided into HPV-associated and HPV-independent, and the latter includes gastric AC, clear cell AC, mesonephroid AC, and endometrioid (among which gastric type is the most common).^[2]^

Gastric-type endocervical adenocarcinomas (GAS) with high malignant degree poor prognosis and insidious development, tend to develop toward the cervical canal and lack specific tumor markers, so it is difficult to diagnose early.^[2]^ We will report a case of GSA which was missed to be diagnosed many times, and discuss its clinical characteristics in order to summarize the experience of diagnosis of GAS. The patient has provided informed consent for the publication of the case.

## 2. Case report

In December 2022, a 46-year-old woman was referred to our hospital, she complained that she had large vaginal transparent discharge without odor for 3 years. Approximately 2 years ago, she was managed at a local community hospital. Then she underwent the ultrasound examination, ThinPrep cytologic test, and HPV genotype test. The ultrasound examination showed that she had multiple uterine leiomyomata. And the results of the ThinPrep cytologic test and the HPV DNA test were negative. Subsequently, she accepted a diagnosis of uterine leiomyomata and was provided with drug therapy with no obvious effects. Subsequently, in July 2017, she underwent colposcopic exam, cervical multipoint biopsy, and endocervical curettage. The pathology suggested the following: (cervical tissue) chronic cervicitis with surface squamous epithelial parakeratosis; (cervical canal) free cervical mucinous glands. For the sake of seeking further diagnosis, the patient went to another hospital. Then she received a pelvic magnetic resonance imaging (MRI) plain scan, which showed the following: uterine leiomyomata, pelvic fluid, and bilateral adnexa cysts. She accepted the ultrasound examination again, which was as follows: intrauterine device, uterine leiomyomata, pelvic fluid, and bilateral ovarian cysts. Then, she underwent uteroscopy examination, diagnostic curettage surgery, and ring extraction. The uteroscopy examination and pathological examination findings based on diagnostic curettage surgery showed no obvious abnormal lesion in the cervix. After that, “laparoscopic resection of uterine fibroids + bilateral salpingectomy” were performed, with “uterine leiomyomata and bilateral chronic salpingitis” indicated by postoperative pathology. The symptoms of vaginal discharge showed improvement within 1 month after the operation. Soon afterward, the discharge volume gradually increased to date.

**Figure 1.**
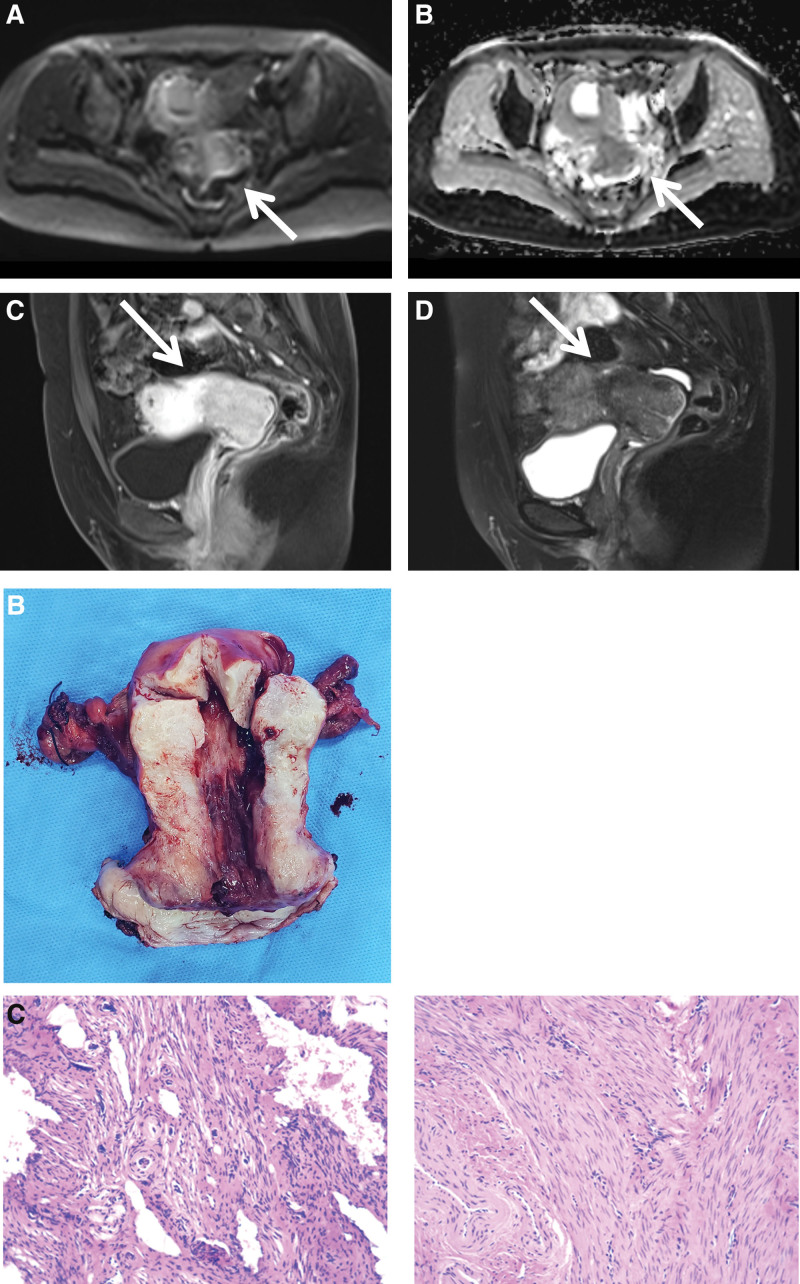
(A) MRI showing abnormal signal of vaginal fornix. (B) Hysterectomy specimen showing cervical tumor. (C) The results of hematoxylin-eosin staining viewed with a microscope (200 magnification). MRI = magnetic resonance imaging.

On November 23, 2022, she visited our hospital for seeking further diagnosis and treatment. Reexamination by gynecological ultrasound examination showed that the uterine isthmus and the anterior and posterior walls of the cervix were thickened, with respect to the possibility of cervical cancer or endometrial carcinoma. The patient had no uterine bleeding, abdominal distention, abdominal pain, urinary frequency, urgency, dysuria, fever, or other discomfort. Her laboratory test results showed no abnormality. By vagino-recto-abdominal examination, we found the following: cervical hypertrophy; enlarged and “barrel-shaped”; no bleeding on touch; qualitative hard; shallowing of left vaginal fornix; and slightly crispation of left cardinal ligament and uterosacral ligament. Her abdominal and pelvic MRI plain scan and diffusion-weighted imaging showed that the signal intensity on T1WI was slightly low and on T2WI was slightly high of the uterine isthmus and cervix which boundaries were still clear and signals were uneven distribution; besides, diffusion was limited slightly in DWI and ADC and the lesions were weak and heterogeneous enhancement in the enhanced scanning involving the vaginal fornix (Fig. 1A). The imaging diagnosis was the following: abnormal signal of vaginal fornix, with the consideration of cervical cancer and the possibility of MR FIGO stage IB3. Considering the final diagnosis depending on the histopathological examination, the patient underwent a diagnostic hysteroscopic resection and intraoperative rapid freezing was also suggestive of cervical adenocarcinoma. Then we talked to the relatives of the patient about the pathologic findings and the result of image analysis, and the relatives of the patient chose “extensive hysterectomy + bilateral ovariectomy + pelvic lymph node resection” (Fig. 1B). Postoperative routine pathological examination results were as follows: invasive cervical adenocarcinoma (non-HPV-related, gastric-type), the lesion infiltrated internal orifice of the uterus, over 5/6 of the cervical wall and nerve; 3 multiple uterine leiomyomata, with the diameter of 0.1 to 0.8 cm; proliferative endometrium; no cancer at bilateral ovaries and parametrium. Postoperative frozen section pathology findings suggested the following: (cervical surface) adenocarcinoma; (posterior vaginal fornix) no cancer; and (left pelvic cavity, right pelvic cavity, adjacent area of left common iliac artery, and adjacent area of right common iliac artery) no cancer in the lymph nodes, which were (0/10; 0/6; 0/1; 0/4), separately. Immunohistochemical stains showed that tumor cells were P16 (+), CEA (+), Ki-67 + (5%), PAX8 (+), PAX2 (−), ER(−), WT-1 (−), GATA-3 (−), MUC5AC (+), MUC6 (+), CK7 (+), CK20 (+, lesion), cdx-2 (+), Vimentin (−), CD10 (+, lesion) (Fig. 1C). In addition, due to the novel coronavirus epidemic, patients postpone postoperative adjuvant chemoradiotherapy.

## 3. Discussion

GAS is the most common HPV-independent adenocarcinoma of the cervix, which was initially described by Japanese pathologists Kojima and his colleagues in 2007.^[3]^ In 2020, WHO classifies endocervical AC according to whether it has HPV infection, of which HPV-independent cervical AC account for 15% in most Western countries.^[4]^ As a special type of cervical adenocarcinoma, the exact incidence rate and global distribution of GAS are still unclear. According to some research, there exist inconsistent reports on the proportion of GAS in cervical adenocarcinoma, with >20% in the Japanese population and 20.8% in the study of Anna et al^[3,5]^ In addition, as for GAS, cytologic examination is difficult and the utility of HPV testing is limited.

GAS is a destructive invasive tumor, presenting at a higher stage, with a high rate of misdiagnosis and missed diagnosis, and poor prognosis. Compared to HPV-associated cervical AC (74%), patients with GSA had a reduced significantly 5-year disease-specific survival rate (38%).^[3]^ GAS has no specific clinical manifestations. A majority of tumors manifest vaginal mucus or watery discharge, pelvic and abdominal mass. However, common symptoms of cervical cancer are uncommon. In addition, the clinical examination of GAS is also not specific, and it appears as a smooth cervix or a thickened and hardened cervix (the so-called “barrel-shaped” cervix). Since its incidence is not related to HPV infection, clinical routine screening methods for cervical cancer are of little value, such as cytological screening and HPV detection. And because of low screening positive rate, the probability of referral to colposcopy is low. Even if patients receive it, the probability of colposcopy finding cervical gastric adenocarcinoma is low, due to the location of the lesions in the cervical canal mostly. Thus, it is necessary for clinicians to be alert to the possibility of GAS when patients perform multiple vaginal discharges but have negative results of HPV and cytology. In our case, the patient has vaginal fluid and no positive findings of HPV and cytology. Although she receives colposcopic exam, cervical multipoint biopsy, endocervical curettage, uteroscopy examination, and diagnostic curettage surgery, neither was confirmed. In summary, early diagnosis of the disease is difficult.

MRI is an auxiliary tool for the early diagnosis of GAS. It is the preferred imaging method for GAS. Because MRI of the tumor has a certain correlation with the pathological findings and it has the ability to evaluate advanced disease by identifying a potential solid component.^[6]^ Besides, MRI could evaluate tumor extension to guide the surgery of advanced cervical adenocarcinoma. Some MRI features of GAS are helpful for diagnosis. The MRI manifestations of GAS are as follows: exogenous or endogenous mass, ill-defined margin, cysts within the tumor, and location in the upper cervical canal or the entire cervix.^[7]^ And T1WI may show low signal intensity, while T2WI shows high signal intensity. In our patient, her abdominal and pelvic MRI showed that the signal intensity on T1WI was slightly low and on T2WI was slightly high of the uterine isthmus, with imaging diagnosis of cervical cancer.

Pathology is the gold standard for the diagnosis of GAS.^[8]^ Histologically, GAS is defined as a tumor with distinct cell borders, whose cytoplasm are clear and/or pale eosinophilic and voluminous, and whose nuclear characteristics include nuclear enlargement, hyperchromasia, and loss of polarity.^[9]^ Immunohistochemistry contributes to the diagnosis of GAS. Immunohistochemically, GAS is generally characterized as follows: negative ER, PR, vimentin, p16, p63, p40, AR, PAX2; positive Trefoil Factor 2 (TFF2), CK7, CEA, and CAIX; positive PAX8, MUC6, HIK1083 (60–80%); positive CK20 and CDX2 (50%); and positive HNF1 (90%).^[4]^ The patient we tested showed P16 (−), PAX8 (+), PAX2 (−), ER (−), MUC6 (+), CK7 (+), CK20 (+, lesion), cdx-2 (+), and Vimentin (−), which are basically identical with the results reported in the relevant documents. The surrogate HPV marker is p16 and HPV-associated uterine cervix commonly shows diffuse p16 positivity. So, in GSA, p16 is usually negative or focally positive. MUC6 and HIK1083 are gastric-type mucin markers, which have an effect on the diagnosis of GSA. But Caterina et al^[10]^ found that the diagnostic accuracy of MUC6 and HIK1083 was not enough. They pointed out that HIK1083 has high specificity but poor sensitivity and MUC6 showed moderate specificity and low sensitivity. Besides, MUC6 can distinguish gastric-type from other mucinous cervical adenocarcinoma.^[11]^ But the double positive of TFF2 and HIK1083 have high specificity for GSA. The reason is that TFF2 is expressed in 80% of GSA but in 12% of non-GSA. Due to the similarity of the immunohistochemical profile between GSA and AC of the pancreatobiliary tract, a PAX8 stain is useful in demonstrating that the source of these tumors is the uterine cervix.^[4]^ CAIX and HNF1 have little help to diagnose GSA. HNF1 can be positive in both GSA and clear cell carcinoma.^[4]^ And CAXI can be expressed in usual-type cervical carcinomas, HPV-associated cervical mucinous carcinomas, and GAS.^[11]^ Therefore, individual immunohistochemical factor has a limited effect on the diagnosis of GSA. Combination of multiple indicators may be of value.

In terms of treatment, there exists controversial and no unified standard. The Chinese Expert Consensus on the Clinical Diagnosis and Treatment of Gastric-type Endocervical Adenocarcinoma (2021 Edition)^[8]^ points out that in view of the high invasiveness of GSA and in the absence of standard treatment methods, the treatment of GAS can refer to Interpretation of the NCCN clinical practice guidelines for cervical cancer (2021.v1) for the treatment of small cell carcinoma. It recommends that locally early patients were mainly treated with surgery, supplemented by radiotherapy and chemotherapy ± targeted therapy after surgery; locally advanced patients underwent concurrent chemoradiotherapy ± targeted therapy. Compared to non-gastric-type endocervical AC, GAS performs poorer progression-free survival (PFS) and overall survival (OS). GAS was resistant to chemotherapy and radiotherapy, which may contribute to poor outcomes. In the treatment of chemotherapy, Shimada et al found that GAS had a lower response rate (46.2% vs 85.0% [17/20], *P* = .048), 5-year PFS rates (38.5% vs 75.0%, *P* = .011, respectively) and OS rates (36.9% vs 90.0%, *P* < .001, respectively) than that of non-gastric-type endocervical AC.^[12]^ In the treatment of chemotherapy, compared to non-gastric-type endocervical AC, the 3-year OS rate and the PFS rate both lower (50.0% vs 78.4%, *P* = .18, 44.4% vs 66.7%, *P* < .05, respectively).^[13]^

## 4. Conclusions

In summary, clinicians still face great challenges in the diagnosis and treatment of GAS. In our case report, the patient visited the local hospital many times and failed to make a definite diagnosis and we diagnosed GSA through the patient’s clinical manifestations, MRI, pathology, and immunohistochemistry. Therefore, for patients with vaginal discharge, cervical canal hypertrophy, and cervical cancer screening negative, clinicians ought to be highly alert to GAS. To avoid misdiagnosis and missed diagnosis and achieve early detection of disease, we should raise awareness of GSA and choose the appropriate treatment to maximize the benefits to patients.

## Author contributions

**Data curation:** Guoyu Jin, Junlei Zhang.

Formal analysis: Qiuju Li.

Supervision: Guoyun Wang, Changzhong Li.

Writing – original draft: Shurong Zhang.

Writing – review & editing: Feier Ding, Chunrun Yang.
